# Transforming tumoroids derived from ALK-positive pulmonary adenocarcinoma to squamous cell carcinoma in vivo

**DOI:** 10.1007/s13577-024-01085-8

**Published:** 2024-06-03

**Authors:** Etsuko Yokota, Miki Iwai, Yuta Ishida, Takuro Yukawa, Masaki Matsubara, Yoshio Naomoto, Hideyo Fujiwara, Yasumasa Monobe, Minoru Haisa, Nagio Takigawa, Takuya Fukazawa, Tomoki Yamatsuji

**Affiliations:** 1 Department of General Surgery, Kawasaki Medical School, Okayama, 700-8505 Japan; 2 General Medical Center Research Unit, Kawasaki Medical School, Okayama, Japan; 3 Department of Pathology, Kawasaki Medical School, Okayama, Japan; 4 Okayama Medical Laboratories Co., Ltd., Kurashiki, Japan; 5 Kawasaki Medical School General Medical Center, Okayama, Japan; 6 Department of Medical Care Work, Kawasaki College of Health Professions, Okayama, Japan; 7 Kawasaki Geriatric Medical Center, Okayama, Japan; 8 Department of General Internal Medicine 4, Kawasaki Medical School, Okayama, Japan

**Keywords:** Lung cancer, *ALK* fusion genes, Adenosquamous carcinoma, Preclinical cancer model, Tumoroids

## Abstract

Approximately 3–5% of non-small cell lung cancers (NSCLC) harbor *ALK* fusion genes and may be responsive to anaplastic lymphoma kinase (ALK) tyrosine kinase inhibitors. There are only a few reports on cell lines with *EML4-ALK* variant 3 (v3) and tumoroids that can be subject to long-term culture (> 3 months). In this study, we established tumoroids (PDT-LUAD#119) from a patient with lung cancer harboring *EML4-ALK* that could be cultured for 12 months. Whole-exome sequencing and RNA sequencing analyses revealed *TP53* mutations and an *EML4-ALK* v3 mutation. PDT-LUAD#119 lung tumoroids were sensitive to the ALK tyrosine kinase inhibitors (ALK TKIs) crizotinib, alectinib, entrectinib, and lorlatinib, similar to NCI-H3122 cells harboring *EML4-ALK* variant 1 (v1). Unexpectedly, clear squamous cell carcinoma and solid adenocarcinoma were observed in xenografts from PDT-LUAD#119 lung tumoroids, indicating adenosquamous carcinoma. Immunostaining revealed that the squamous cell carcinoma was ALK positive, suggesting a squamous transformation of the adenocarcinoma. Besides providing a novel cancer model to support basic research on ALK-positive lung cancer, PDT-LUAD#119 lung tumoroids will help elucidate the pathogenesis of adenosquamous carcinoma.

**Supplementary Information:**

The online version contains supplementary material available at 10.1007/s13577-024-01085-8.

## Introduction

In recent years, the identification of lung cancers harboring rare driver mutations has increased, leading to a considerable increase in the number of approved targeted therapies. This approach has substantially improved the prognosis of patients with lung cancer [1]. Anaplastic lymphoma kinase (ALK) rearrangements are detected in 3–5% of all non-small cell lung cancers (NSCLCs), and ALK tyrosine kinase inhibitors (TKIs) improve the prognosis of patients with ALK-positive NSCLC [2]. *EML4* is located on chromosome 2p21 and contains 26 exons. There are various fusion breakpoints in multiple exons of *EML4* in *EML4-ALK-*positive lung cancer. Among these, *EML4-ALK* variant 1 (v1), where exons 20–29 of *ALK* fuse with exons 1–13 of *EML4*, and variant 3 (v3), where exons 20–29 of *ALK* fuse with exons 1–6 of *EML4*, are the most commonly observed variants, constituting approximately 75–80% of the total variants [3]. Furthermore, patients with lung cancer harboring *EML4-ALK* v3 or *TP53* mutations have a poor prognosis [4, 5].

In recent years, organoid research has been conducted using pluripotent stem cells and biopsy and surgical specimens [6, 7]. In cancer research, organoids have emerged as novel preclinical models derived from various malignant tumors, serving as alternatives to traditional two-dimensional cell cultures and genetically engineered mouse models [8]. In our previous studies, we have highlighted the clinical application of lung tumoroids derived from surgically removed lung cancer tissues and malignant pleural effusions, demonstrating their utility in personalized medicine [9]. Although several cell lines have been used in the study of *EML4-ALK-*positive lung cancer, the number of cancer models remains limited. There is a need to develop preclinical cancer models that further reflect the characteristics of patient tumors to analyze their pathogenesis and develop therapies for these types of tumors.

In the present study, we established tumoroids, PDT-LUAD#119, from a patient with NSCLCs harboring *EML4-ALK* v3, characterized the tumoroids, and evaluated their utility as a preclinical model. We also discuss the adenocarcinoma-to-squamous cell carcinoma transformation observed in an in vivo xenograft tumor derived from PDT-LUAD#119 lung tumoroids.

## Materials and methods

### Cell lines and their culture conditions

NCI-A549 pulmonary adenocarcinoma cells harboring *KRAS*^G12S^ and NCI-H2228 pulmonary adenocarcinoma cells harboring variant 3a and 3b (v3a/b) *EML4-ALK* fusion were obtained from the American Type Culture Collection (Manassas, VA, USA). NCI-H3122 pulmonary adenocarcinoma cells harboring variant 1 (v1) *EML4-ALK* fusion were sourced from the National Institute of Health (NIH) (Rockville, MD, USA). These cells were cultured as monolayers in the Roswell Park Memorial Institute (RPMI) 1640 medium for NCI-H2228 and NCI-H3122, or in Dulbecco’s modified Eagle medium (DMEM) for NCI-A549. The media were supplemented with 10% heat-inactivated fetal bovine serum, 100 μg/mL streptomycin, and 100 units/mL penicillin. All cells were maintained at 37 °C in an atmosphere of 5% CO_2_, authenticated using short tandem repeat analysis, and regularly tested for *Mycoplasma* contamination using the TaKaRa PCR *Mycoplasma* Detection Set (Takara Bio, Inc., Otsu, Japan).

### Patient-derived tumoroid culture

Patient-derived lung adenocarcinoma (LUAD) tumoroids (PDT-LUAD#119) were developed using tumoroid culture systems, as described previously [9]. The research protocol was approved by the Ethics Committee of the Kawasaki Medical School (reference number 3171-5). All participating patients signed an informed consent form that was authorized by the relevant authority.

### Next-generation and Sanger sequencing

Next-generation sequencing (whole-exome sequencing and RNA sequencing) was conducted as previously described [9]. Sanger sequencing was conducted by Eurofins Genomics K. K. (Tokyo, Japan) using the following primers: for TP53 Exon 4, 5′-CAAGCAATGGATGATTTGATGCTGTC-3′ and 5′-TAGGTTTTCTGGGAAGGGACAGAAGATG-3′; and for TP53 Exon 10, 5′-ACTAAATGCATGTTGCTTTTGTACCGTCA-3′ and 5′-CAGGATGAGAATGGAATCCTATGGCTTT-3′.

### Reverse transcription-polymerase chain reaction (RT-PCR)

Total cDNA of lung cancer cells or lung tumoroids was synthesized using reverse transcription (RT) with the PrimeScript™ RT reagent Kit (Takara Bio, Shiga, Japan). PCR was used to amplify the fusion point of EML4-ALK v1 and v3 mRNA using the primers 5′-GAAAATTCAGATGATAGCCGTAATAAATTGTCGAA-3′ and 5′-GTCTTGCCAGCAAAGCAGTAGTTGGGGTTGTAGT-3′. The primer pair used for the amplification of *ACTB* mRNA was 5′-AGAGAGGCATCCTCACCCTGAAGT-3′ and 5′-GATAGCACAGCCTGGATAGCAACG-3′.

### Fluorescence in situ hybridization (FISH)

Alpha-satellite DNA for all chromosomes was produced as previously described [9] and labeled using Nick Translation Mix (Sigma-Aldrich, St. Louis, MO, USA) with rhodamine (orange). EML4 and ALK probes were obtained from CytoTest (Rockville, MD, USA). A standard protocol was utilized to conduct FISH [10], and the samples were examined under a fluorescence microscope (ECLIPSE Ni, DS-Qi2; Nikon, Tokyo, Japan).

### Immunoblotting and immunohistochemistry

Immunoblot analysis and immunohistochemistry were carried out according to established protocols [11]. The primary anti-NKX2-1 antibody (8G7G3/1) was purchased from DAKO (Carpinteria, CA, USA) for immunohistochemical staining. The p40 antibody (Cat. ABS552/ MABS519 11F12.1) used for immunohistochemical staining was obtained from Merck Millipore (Burlington, MA, USA). The primary anti-ALK antibody (D5F3) used for immunohistochemical staining and immunoblotting was purchased from Cell Signaling Technology (Danvers, MA, USA).

### Xenograft inoculation of lung tumoroids

Cells from PDT-LUAD#119 lung tumoroids (4.0 × 10^6^ cells) were dissociated with TrypLE™ Express Enzyme (Thermo Fisher Scientific), and then combined with 50 μL of basement membrane extract type 2 (BME type 2) and subsequently injected subcutaneously into 5-week-old NOD/Shi-*scid*/IL-2Rγnull (NOG) mice (Charles River Laboratories Japan, Atsugi, Japan). The mice were euthanized when the subcutaneous tumor diameter reached 15 mm. The duration from xenograft initiation to euthanasia was approximately 120 days. All experimental procedures were approved by the Animal Research Committee of Kawasaki Medical School (Reference Number: 23-047), and animal care and handling were conducted in accordance with committee regulations.

### Luminescence cell viability assay

Tumoroids were enumerated and suspended in BME type 2; 4-μL droplets were seeded in clear-bottom, white-walled flat-bottom 96-well culture plates (PerkinElmer, Waltham, MA, USA), and then medium was added. Twenty-four hours post-seeding, ALK TKI inhibitors were added to the medium. Viability assessment was conducted 72 h post-treatment using the Celltiter-Glo^R^ 2.0 Cell Viability Assay (Promega, Madison, WI, USA), following the manufacturer's instructions. Luminescence readings were obtained using a Varioskan LUX multimode microplate reader (Thermo Fisher Scientific, Rockford, IL, USA).

## Results

### Clinical and pathological presentation of the patient harboring the parental lung cancer of PDT-LUAD#119

**Fig. 1:**
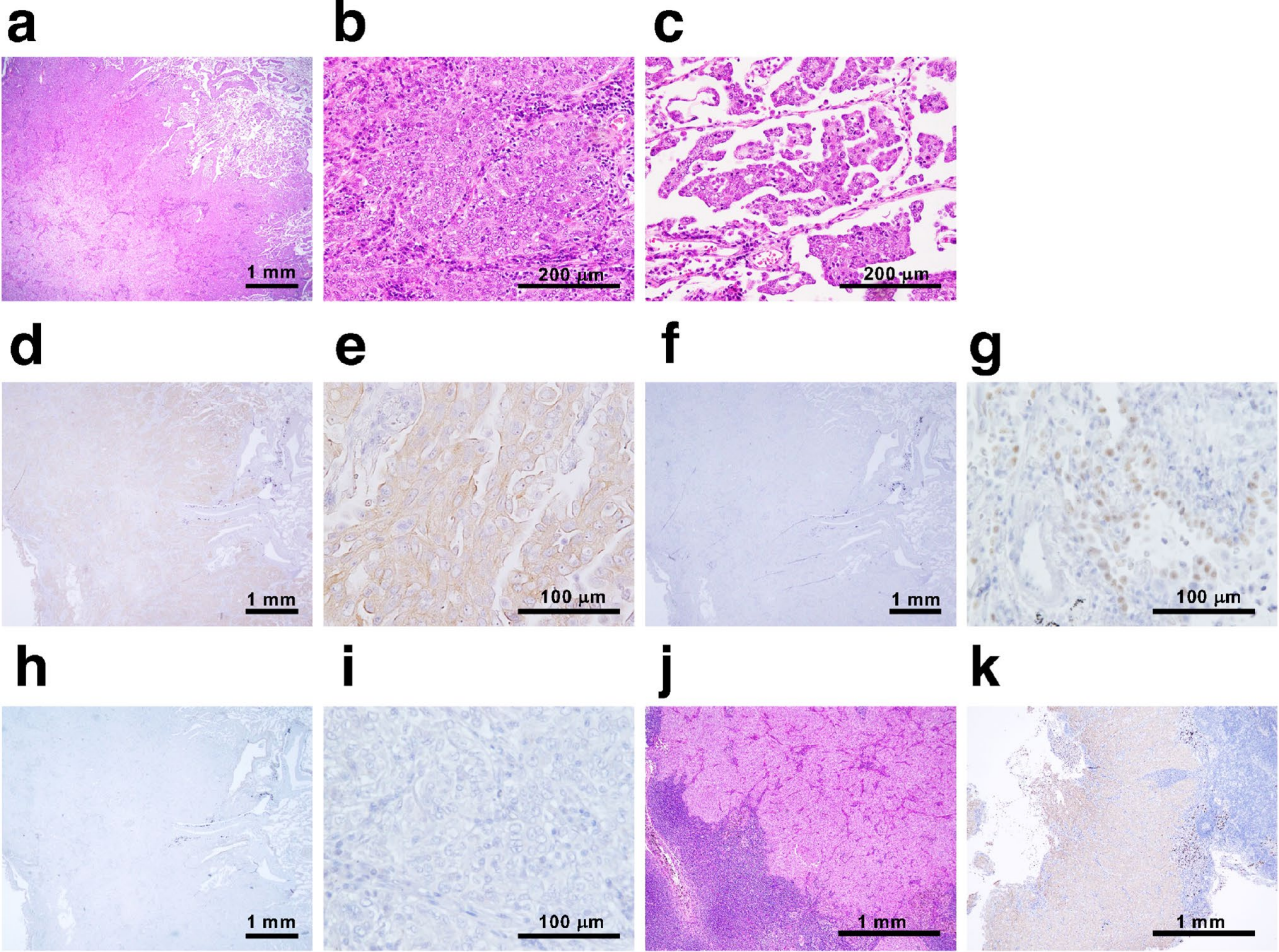
Pathological findings of the primary tumor. **a** Hematoxylin and eosin (HE)-stained tissue section of the parental lung cancer, revealing distinctive pathological features of solid adenocarcinoma. **b**, **c** Micropapillary adenocarcinoma was observed at the periphery of the tumor. Immunohistochemical staining showed widespread expression of ALK in the tumor (**d**), and the expression was localized to the cytosol (**e**). **f**, **g** Partial expression of NKX2-1 was observed in the tumor. **h**, **i** No p40 expression was observed. **j**, **k** ALK-positive metastasis was observed in inferior mediastinum lymph node station 7

An 87-year-old woman who fell into a ditch and had difficulty walking on her own was admitted to the emergency room (ER) of our hospital. In addition to a bruise on her left buttock, a nodule was detected in the upper lobe of the right lung on computed tomography (CT) (Supplementary Fig. 1a). Following a thorough examination, the patient was suspected to have lung cancer (Supplementary Fig. 1b, cT1cN0M0 stage IA3), and right lower lobectomy via video-assisted thoracic surgery (VATS) was performed. Pathological examination revealed that the size of the tumor was 22 mm × 18 mm (Supplementary Fig. 1c and 1d), and it was diagnosed as solid-predominant pulmonary adenocarcinoma mixed with micropapillary adenocarcinoma (Fig. 1a–c). No *EGFR* mutations were found; however, ALK expression was detected in the cytosol based on immunohistochemical staining using anti ALK antibody (clone D5F3; Supplementary Fig. 1e, Fig. 1d, e). Nuclear NKX2-1 expression was partially detected (Fig. 1f, g); however, no p40 expression was observed in the primary tumor (Fig. 1h, i). These findings, including the results of hematoxylin and eosin staining, suggested that the primary tumor was pulmonary adenocarcinoma with negligible amounts of squamous cell carcinoma components. The tumor cells had metastasized to the hilar and mediastinal lymph nodes, resulting in a final diagnosis of stage IIIA (pT1N2M0, Fig. 1j, k).

### Generation tumoroids derived from a lung cancer patient with ALK-positive pulmonary adenocarcinoma

**Fig. 2:**
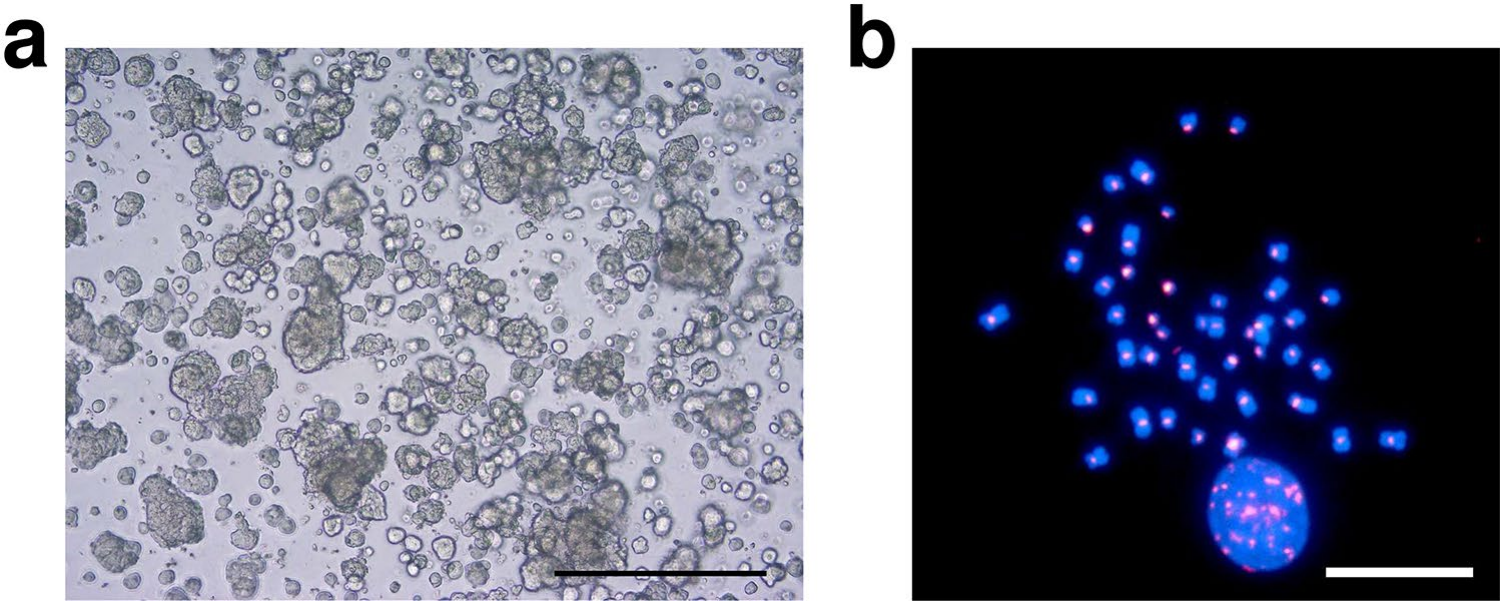
Patient-derived tumoroids: PDT-LUAD#119 derived from a patient with lung cancer harboring EML4-ALK v3a/b.** a** Representative bright-field microscopy images (left ternate columns) of PDT-LUAD#119 lung tumoroids established with different media. Scale bar 500 μm. **b** A metaphase FISH image (right columns) of PDT-LUAD#119 lung tumoroids. Scale bar 20 μm

We successfully generated lung tumoroids (PDT-LUAD#119) from surgical specimens of the patient upon culture in five out of six media (AO; AO with nutlin-3a; AO without FGF-7, FGF-10, R-spondin-1 and Noggin; Shi-Tsao; and Shi-Tsao with nutlin-3a; Table 1) [9, 12–14]. The established tumors were dense and irregular, as is often observed in cancer organoids, regardless of the establishment conditions (Fig. 2a). We added an MDM-2 inhibitor (nutlin-3a, which activates wild-type p53) to the media to inhibit the growth of organoids from normal lung epithelial cells [15]. We performed the following analyses using tumoroids established with AO and nutlin-3a (AO w/ nutlin-3a). No morphological differences were observed among the culture conditions (data not shown). The FISH analysis performed using an alpha-satellite probe for karyotyping unveiled aneuploid karyotype (2*n* = 43) in PDT-LUAD#119 lung tumoroids, confirming their successful establishment in lung cancer (Fig. 2b). In subsequent experiments, we used PDT-LUAD#119 lung tumoroids, established with AO w/ nutlin-3a.

**Table 1:** Lung tumoroid media

Lung tumorioid media	Publications	
AO	Sachs, N. et al. EMBO J. 38, embj.2018100300 (2019)	◯
AO with nutlin-3a	Sachs, N. et al. EMBO J. 38, embj.2018100300 (2019)	**◯**
AO without FGF-7, FGF-10, R-spondin-1 and Noggin	Yokota, E et al. NPJ Precis Oncol. 5:29. https://doi.org/10.1038/s41698-021-00166-3. (2021)	**◯**
MBM	Kim, M. et al. Nat. Commun. 10, 3991 (2019)	X
Shi-Tsao	Shi, R. et al. Clin. Cancer Res. 26,1162–1174 (2019)	**◯**
Shi-Tsao with nutlin-3a		**◯**

◯: PDT-LUAD#119 lung tumoroids were succcessfully establised

### Whole-exome and RNA sequencing analysis of PDT-LUAD#119 lung tumoroids

**Fig. 3:**
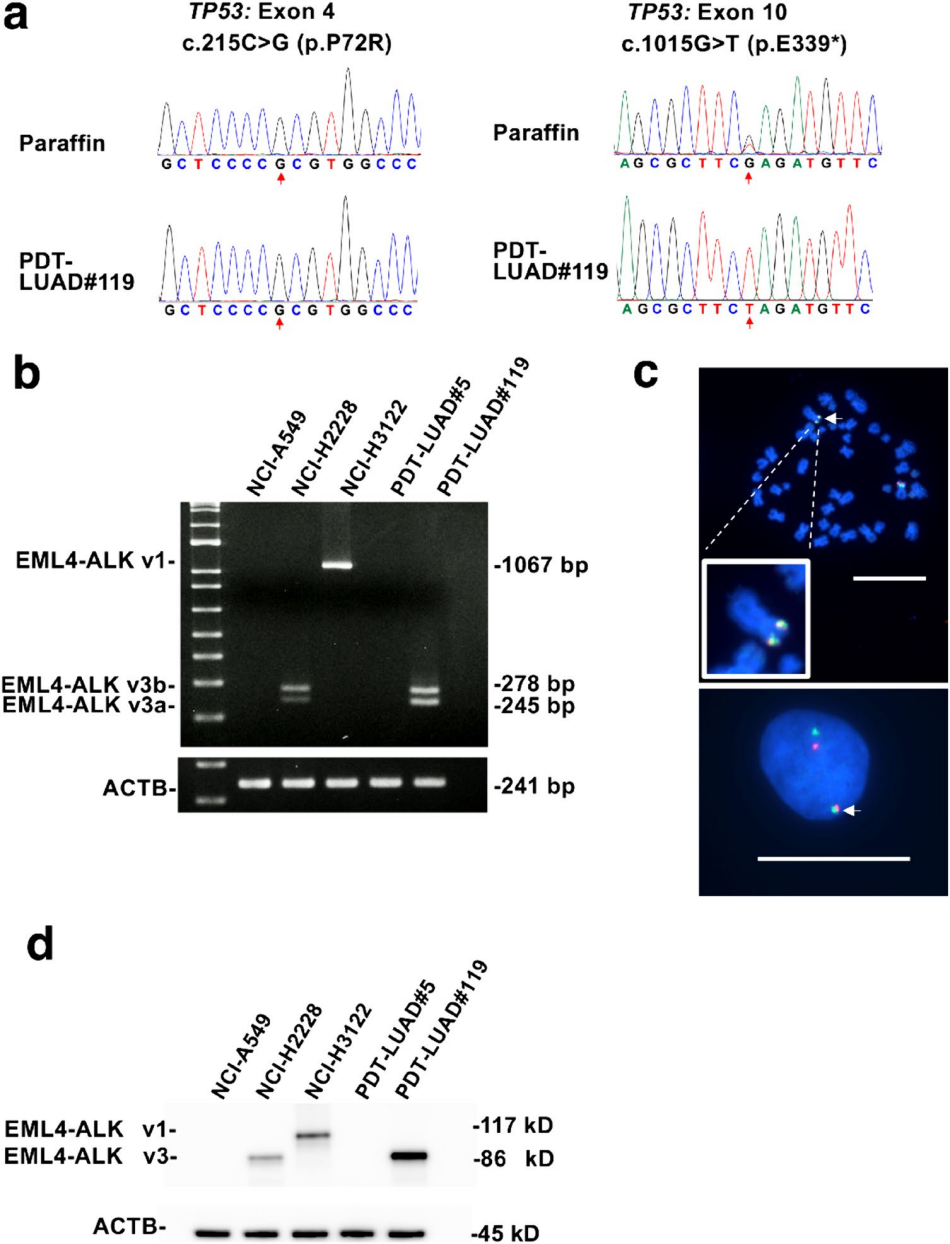
PDT-LUAD#119 lung tumoroids carry *EML4* -*ALK* v3. **a** Sanger sequencing confirmed the *TP53* mutations observed in DNA from paraffin-embedded parental tissue and PDT-LUAD#119 lung tumoroids. **b** Detection of fusion cDNAs linking exon 6 of *EML4* to exon 20 of *ALK* using RT-PCR. Two RT-PCR products of 278 bp (corresponding to v3b) and 245 bp (corresponding to v3a) were observed in NCI-H2228 pulmonary adenocarcinoma cells and PDT-LUAD#119 lung tumoroids. NCI-H3122 pulmonary adenocarcinoma cells had 1067-bp products corresponding to v1 according to RT-PCR. *ACTB* was used as the internal control. **c** FISH analysis of representative cells (upper column, metaphase; lower column, interphase) in PDT-LUAD#119 lung tumoroids with differentially labeled probes for EML4 (green) and ALK (orange). Two fusion signals are present in the merged image (arrows). **d** Immunoblotting detected EML4-ALK v1 in NCI-H3122 pulmonary adenocarcinoma cells and EML4-ALK v3a/b in NCI-H2228 pulmonary adenocarcinoma cells and PDT-LUAD#119 lung tumoroids. ACTB was used as a control

We subsequently analyzed the principal lung cancer genetic variants in PDT-LUAD#119 lung tumoroids, including the *ALK* fusion gene, using next-generation sequencing (NGS; please refer to the data availability statement). PDT-LUAD#119 lung tumoroids showed two types of pathogenic mutations in *TP53* (c.215C>G, p.P72R) and *TP53* (1015G>T, p.E339*), which were confirmed using Sanger sequencing of the genome extracted from the patient’s tumor (Fig. 3a). RNA sequencing detected EML4-ALK v3a/b in the RNA extracted from the tumoroids, which was confirmed using RT-PCR with specific primers. A pair of 278- and 245-bp PCR products, corresponding to *EML4-ALK* v3b and v3a, respectively, was detected in NCI-H2228 pulmonary adenocarcinoma cells harboring *EML4-ALK* v3a/b [16] and PDT-LUAD#119 lung tumoroids using the primer set for amplification of the *EML4*-*ALK* fusion cDNA (Fig. 3b). These two specific products were not observed in NCI-A549 pulmonary adenocarcinoma cells harboring *KRAS*^*G12S*^, NCI-H3122 pulmonary adenocarcinoma cells harboring *EML4-ALK* v1, and PDT-LUAD#5 lung tumoroids with *BRAF*^*G469A*^. In NCI-H3122 pulmonary adenocarcinoma cells, analysis using the above primers detected a PCR product of 1067 bp. The FISH analysis detected the *EML4-ALK* fusion gene in PDT-LUAD#119 lung tumoroids (Fig. 3c). Furthermore, the immunoblot analysis showed that EML4-ALK v1 was present in NCI-H3122 pulmonary adenocarcinoma cells, and EML4-ALK v3a/b was present in NCI-H2228 pulmonary adenocarcinoma cells and PDT-LUAD#119 lung tumoroids (Fig. 3d).

### PDT-LUAD#119 lung tumoroids were sensitive to ALK TKIs

**Fig. 4:**
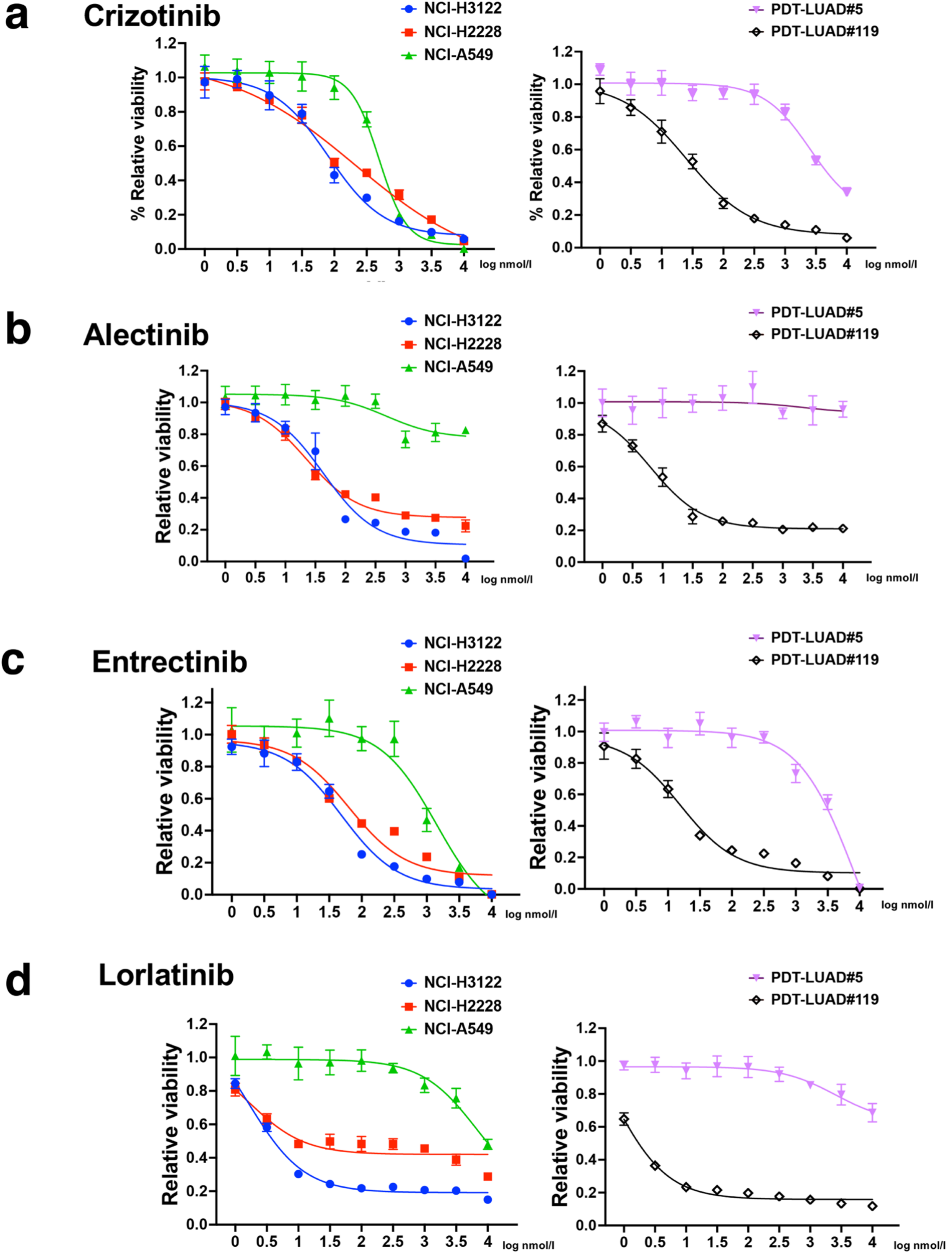
Sensitivity of PDT-LUAD#119 lung tumoroids to ALK TKIs. Dose responses of NCI-H3122 cells (blue), NCI-H2228 cells (red), NCI-A549 cells (green), PDT-LUAD#5 lung tumoroids (violet), and PDT-LUAD#119 lung tumoroids (black) after treatment with (**a**) crizotinib, (**b**) alectinib, (**c**) entrectinib, and (**d**) lorlatinib. Cell viability assay of tumoroids was conducted 72 h after treatment. Data are presented as mean ± SD of six independent experiments

We analyzed the viability of PDT-LUAD#119 lung tumoroids in addition to the other types of tumoroids and lung cancer cells used in this study. NCI-A549 pulmonary adenocarcinoma cells harboring *KRAS*^G12S^ and PDT-LUAD#5 lung tumoroids harboring *BRAF*^*G469A*^ were resistant to all four ALK TKIs (Fig. 4a–d). Crizotinib and entrectinib at higher concentrations inhibited the growth of NCI-A549 cells and PDT-LUAD#5 lung tumoroids compared to the other two kinds of ALK TKIs (alectinib and lorlatinib). Among lung cancer cells, NCI-H3122 cells with *EML4-ALK* v1 tended to be more sensitive to ALK TKIs than NCI-H2228 cells with *EML4-ALK* v3a/b. This finding is consistent with previous reports showing that lung cancers with *EML4-ALK* v3a/b are less sensitive to ALK TKIs than those with *EML4-ALK* v1 mutations. In contrast, PDT-LUAD#119 lung tumoroids showed relatively high sensitivity to ALK TKIs despite having *EML4-ALK* v3a/b.

### Xenografts derived from PDT-LUAD#119 lung tumoroids were diagnosed as adenosquamous carcinoma rather than pulmonary adenocarcinoma

**Fig. 5:**
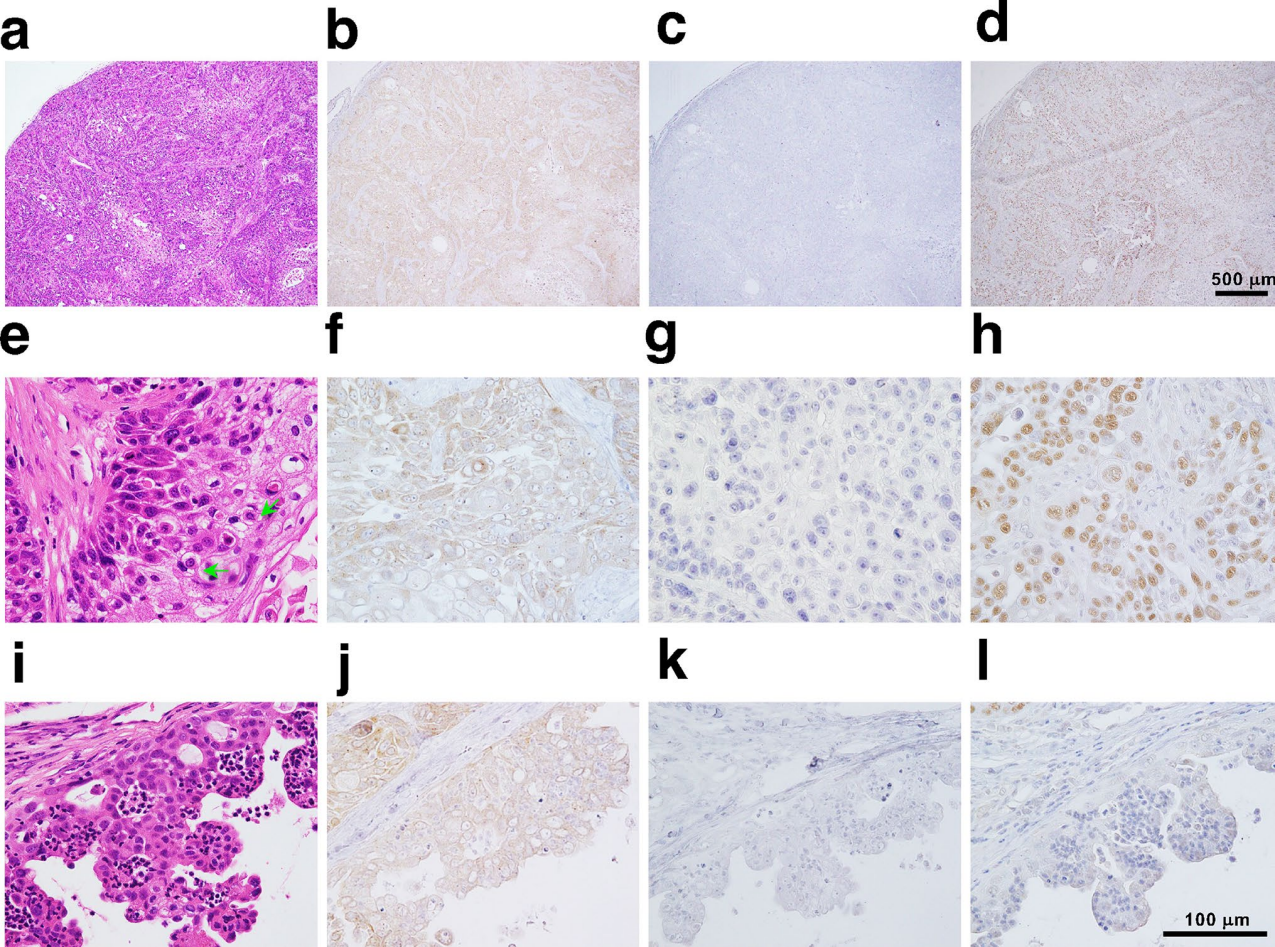
Transformation of pulmonary adenocarcinoma tumoroids with EML4-ALK into adenosquamous carcinoma in vivo. Distinctive pathological features of squamous cell carcinoma was observed in xenografts derived from PDT-LUAD#119 lung tumoroids (**a**–**h**). Immunohistochemical analysis showed extensive ALK (**b**, **f**) and p40 (**d**, **h**) expression in the tumor; however, no NKX2-1 expression was observed (**c**, **g**). In addition to keratinization and abundant eosinophilic cytoplasm, intercellular bridges (green arrows) were observed (**e**). Adenocarcinoma was observed in some parts (**i**–**l**). ALK expression was observed in the adenocarcinoma portion (**j**), whereas no NKX2-1 and little p40 expression were observed in the area (**k**, **l**). Scale bar 500 μm (**a–d**), 100 μm (**e–l**)

To explore if the established tumoroids faithfully replicated the parental lung cancer pathology in vivo, patient-derived xenografts (PDXs) were established in NOD/Shi-*scid*/IL-2Rγnull (NOG) mice through subcutaneous inoculation of PDT-LUAD#119 lung tumoroids. Unexpectedly, most xenografts from PDT-LUAD#119 lung tumoroids showed squamous cell carcinoma (Fig. 5a–h) mixed with adenocarcinoma in some parts (Fig. 5i–l) and finally diagnosed as adenosquamous carcinoma. Importantly, ALK expression was broadly positive, including the areas with squamous cell carcinoma (Fig. 5b, f and j). Although NKX2-1 expression was not detected (Fig. 5c, g), p40 expression was broadly observed in the xenografts (Fig. 5d, h). These results suggest that *EML4-ALK* v3-harboring pulmonary adenocarcinoma tumors might transform into squamous cell carcinoma during tumor formation in vivo.

## Discussion

Yoshida et al. [17] reported the pathological features of ALK-positive lung cancer based on the examination of 54 patients who underwent surgical resection. They reported two distinctive findings in most ALK-positive tumors (78%): solid signet cell patterns and mucinous cribriform patterns, at least locally. In contrast, these features are rare in tumors with wild-type ALK (1%) [17]. In our case, these characteristic initial findings of ALK-positive lung cancer were not observed in primary lung cancer. These features were also observed in lung cancers with other fusion genes [18]. Therefore, future investigations may allow for the estimation of genetic subtypes based on histological findings.

Previous reports indicate that patients with *EML4-ALK* v1 show a better clinical response to ALK TKIs (such as crizotinib or alectinib) than patients with other types of ALK fusion variants [3]. In contrast, patients with *TP53* mutations and *EML4-ALK* v3 show a particularly poor prognosis [5]. However, PDT-LUAD#119 lung tumoroids harboring *EML4-ALK* v3 and *TP53* mutations were as sensitive to ALK TKI as NCI-H3122 cells with *EML4-ALK* v1 and *TP53* mutations in the present study. Therefore, a response to an ALK-TKI may be expected if a patient from whom a tumor was established relapses with lung cancer.

In this study, a tumor from a patient with pulmonary adenocarcinoma carrying *EML4-ALK* v3 unexpectedly formed squamous cell carcinoma and adenocarcinoma after inoculation into NOG mice and was finally diagnosed as pulmonary adenosquamous carcinoma. According to WHO Classification 5th edition, pulmonary adenosquamous carcinoma is a cancerous tumor composed of squamous cell carcinoma and adenocarcinoma components, with each component accounting for over 10% of the entire tumor [19]. One of the main mechanisms underlying the development of adenosquamous carcinoma is its transformation into squamous cell carcinoma. Adenosquamous carcinoma shares driver genes with adenocarcinoma but does not share them with squamous cell carcinoma. This finding suggests differentiation from adenocarcinoma to adenosquamous carcinoma, but not vice versa [20, 21]. Additionally, studies using genetically engineered mouse models indicate that the deletion of *LKB1* transforms lung adenocarcinoma into squamous cell carcinoma [22]. A limitation of the study is that only one type of tumoroid derived from a single patient with ALK-positive lung cancer was established. Therefore, further research is needed to determine the mechanism underlying the transformation of pulmonary adenocarcinoma carrying EML4-ALK into pulmonary adenocarcinoma and squamous cell carcinoma in mice.

There are only a few models for the carcinogenesis of adenosquamous carcinoma, and the PDT-LUAD#119 xenograft may serve as a suitable model for analyzing the mechanisms underlying the development of adenosquamous carcinoma. Our results raised issues regarding the usefulness of tumoroids as preclinical models.

### Supplementary Information

Below is the link to the electronic supplementary material.

Supplementary file1 (EPS 42907 kb)

## Data Availability

Raw sequencing data for the exome sequences can be accessed under controlled conditions at the Japanese Genotype–Phenotype Archive (JGA) (accession code JGAS000664) for general research use. Access to these data requires an application through the National Bioscience Database Center (NBDC). Interested parties may obtain direct access from the authors to any additional relevant data. After the publication of this paper, we intend to make the PDT-LUAD#119 lung tumoroids available through the RIKEN Cell Bank (https://cell.brc.riken.jp/en/).
